# Could martial arts fall training be safe for persons with osteoporosis?: a feasibility study

**DOI:** 10.1186/1756-0500-3-111

**Published:** 2010-04-22

**Authors:** Brenda E Groen, Ellen Smulders, Jacques Duysens, Wim van Lankveld, Vivian Weerdesteyn

**Affiliations:** 1 Sint Maartenskliniek Research, Development and Education, Hengstdal 3, 6522 JV Nijmegen, the Netherlands; 2 Institute for Fundamental and Clinical Human Movement Sciences, Vrije Universiteit, Van der Boechorststraat 9, 1081 BT Amsterdam, the Netherlands; 3 Research Centre for Movement Control and Neuroplasticity, Department of Biomedical Kinesiology, Tervuursevest 101, 3001 Leuven, Belgium; 4 Sint Maartenskliniek, Department of Rheumatology, Hengstdal 3, 6522 JV Nijmegen, the Netherlands; 5 Radboud University Nijmegen Medical Centre, Department of Rehabilitation, Geert Grooteplein-Zuid 10, 6500 HB Nijmegen, the Netherlands

## Abstract

**Background:**

Osteoporosis is a well-established risk factor for fall-related hip fractures. Training fall arrest strategies, such as martial arts (MA) fall techniques, might be useful to prevent hip fractures in persons with osteoporosis, provided that the training itself is safe. This study was conducted to determine whether MA fall training would be safe for persons with osteoporosis extrapolated from the data of young adults and using stringent safety criteria.

**Methods:**

Young adults performed sideways and forward MA falls from a kneeling position on both a judo mat and a mattress as well as from a standing position on a mattress. Hip impact forces and kinematic data were collected. For each condition, the highest hip impact force was compared with two safety criteria based on the femoral fracture load and the use of a hip protector.

**Results:**

The highest hip impact force during the various fall conditions ranged between 1426 N and 3132 N. Sideways falls from a kneeling and standing position met the safety criteria if performed on the mattress (max 1426 N and 2012 N, respectively) but not if the falls from a kneeling position were performed on the judo mat (max 2219 N). Forward falls only met the safety criteria if performed from a kneeling position on the mattress (max 2006 N). Hence, forward falls from kneeling position on a judo mat (max 2474 N) and forward falls from standing position on the mattress (max 3132 N) did not meet both safety criteria.

**Conclusions:**

Based on the data of young adults and safety criteria, the MA fall training was expected to be safe for persons with osteoporosis if appropriate safety measures are taken: during the training persons with osteoporosis should wear hip protectors that could attenuate the maximum hip impact force by at least 65%, perform the fall exercises on a thick mattress, and avoid forward fall exercises from a standing position. Hence, a modified MA fall training might be useful to reduce hip fracture risk in persons with osteoporosis.

## Background

Hip fractures among the elderly are a health problem associated with high mortality and morbidity rates. In particular persons with osteoporosis or low bone mineral density (BMD) are at risk for hip fractures due to their reduced bone strength [1,2]. Therefore, in clinical practice hip fracture prevention focuses mainly on treating osteoporosis.

About 90% of hip fractures are caused by falls [3]. Apart from a low BMD, fall characteristics have been identified as independent risk factors for hip fractures [1,2]. Hence, fall prevention and reduction of fall severity may also prevent hip fractures. Falls with the highest risk for hip fractures are sideways falls and falls with direct impact on the greater trochanter of the proximal femur [2]. To reduce the hip fracture risk in these types of falls, hip protectors may be useful. In vitro experiments have shown that the best hip protectors can attenuate femoral impact forces by as much as 85% [4,5]. However, to prevent hip fractures in everyday life, user compliance is a problem [6].

Alternatively, people may be taught safe fall arrest strategies. Recent studies have indicated that fall strategies based on martial arts (MA) fall techniques reduce the impact forces during a volitional fall. When using an MA fall technique, the fall is changed into a rolling movement. During the roll the forces are distributed over a larger impact site. Furthermore, the amount of energy to be absorbed during impact is reduced because kinetic energy is preserved during the rolling movement. Experimental studies have shown that MA techniques during a volitional fall reduce hip impact forces, which presumably reduces the hip fracture risk as well [7-9]. Recently, MA fall training that consisted of 5 weekly training sessions of 45 minutes was included in a successful falls prevention program for healthy elderly persons [10]. A further experimental study revealed that older participants were able to learn the MA techniques during the five weekly training sessions; the improved performance reduced the hip impact force during a volitional fall [11]. For safety reasons, persons with osteoporosis have been excluded from these fall training studies. However, persons with osteoporosis are expected to experience the most benefits from such training because of their high fracture risk if they fall.

The purpose of the present study was to determine whether MA fall training is safe for persons with osteoporosis. For obvious safety reasons, this could not be directly assessed using persons with osteoporosis. Therefore, we measured the hip impact forces during the MA fall exercises from a kneeling and a standing position onto both a judo mat and a thick mattress in a group of young adults. We focused on sideways and forward falls, as these falls have the highest risk for direct hip impact and hip fractures. To determine whether the impact forces are within the safety limits for persons with osteoporosis, two safety criteria were defined based on the femoral fracture load in elderly women [12]. It was hypothesized that for persons with osteoporosis practicing falls from a kneeling position are only safe if performed on a thick mattress while falls from a standing position are never safe.

## Methods

### Participants

Healthy, young individuals without prior experience in MA fall techniques participated in this study. Six participants (age: 23-44 years, weight: 57-85 kg, height: 1.74-1.86 m) performed the MA fall training on a judo mat and six participants (age: 23-44 years, weight: 55-73 kg, height: 1.71-1.86 m) performed the training on a thick mattress. All participants signed informed consent prior to participation. The Ethical Board for the region Arnhem-Nijmegen approved the protocol (2004/152).

### Fall training

**Figure 1:**
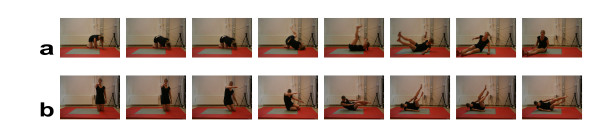
**Photo series of martial arts fall techniques from a kneeling position**. a) Martial arts fall techniques during a forward fall and b) during a sideways fall from kneeling position on the judo mat.

Each participant received individual fall training for approximately two hours. The fall exercises that were performed were the sideways and forward fall techniques as included in the Nijmegen Falls Prevention Program [10]. The three most important characteristics of MA techniques are the rolling movement, head protection by neck flexion and the use of the arm to stop the rolling movement. In forward falls, trunk flexion and rotation enable participants to roll over the scapula of the ipsilateral shoulder and diagonally across the back to the contralateral hip region (Figure 1a). In sideways falls, participants roll over the ipsilateral hip to the scapula of the ipsilateral shoulder; this is achieved by flexion, lateral flexion and rotation of the trunk (Figure 1b). Both sideways and forward fall exercises started in a sitting position; these were not measured since they were assumed to have no hip fracture risk. Thereafter, falls from kneeling and standing positions followed. The fall exercises were performed either on the judo mat (4 cm thick polyurethane foam, size 1.2 × 1.2 m) or on the 25 cm thick gymnasium mattress (size 2.5 × 1.25 m). Each fall condition was performed for at least 8 trials.

### Data collection

During the trials, force data were collected by a force plate (1.2 × 1.2 m, Bertec Corporation, Columbus, Ohio, USA) at a sample rate of 2400 Hz, which was embedded in a 2.2 × 2.7 m platform and covered with judo mats or the thick mattress. It must be emphasized that the judo mat or mattress was not supported by any other surface than the force plate. Hence, all (vertical) forces that are applied to the mat are measured by the force plate. Similar set-ups are used by other groups, for instance, for mechanical testing of the force-attenuating effects of low stiffness floors on peak impact forces on the skin surface of the greater trochanter of the femur and the femoral neck [13]. A 6-camera 3D motion analysis system (Primas, Delft University of Technology, Delft, The Netherlands) was used to collect the 3D positions of reflective markers at 100 Hz. The markers were attached bilaterally to the wrist, elbow, acromion and the pelvis. Kinematic and force data were collected synchronously.

### Data analyses

For all falls from kneeling and standing positions, hip impact forces were determined. Hip impact force was, in general, the first distinct peak in the force curve after fall initiation. Kinematic data were used to confirm whether indeed this peak corresponded to hip impact using the vertical position of the markers. For each fall condition, the maximum vertical hip impact force was determined for each participant (Fmax). For each fall exercise the highest Fmax observed during all the trials of all participants was used to assess the safety of the fall training.

### Safety criteria

Two safety criteria were constructed to determine which fall exercises and conditions were considered to be safe for persons with osteoporosis. Both safety criteria were based on the femoral fracture load of elderly women. Following Kannus and coworkers [4], we used the mean femoral fracture load of 3100 N (SD 1200 N) as determined for cadaveric femora of a group of elderly women by Cheng and coworkers [12].

The first safety criterion implied that the femoral load during a fall exercise should not exceed the average fracture load for elderly women minus 2 SD (700 N: 3100 N - 2 * 1200 N). In other words, the threshold was set at a value that should be safe for 97.7% of the elderly women. Because the femoral load is not equal to the external hip impact force as measured with the force plate, we took two mediating factors into account. Firstly, we included the expected protective effects of soft tissue around the hip. The mean attenuation of the peak impact force caused by soft tissue is 13% in elderly women [14]. Because persons with osteoporosis often have relatively little adipose tissue, we used a 10% reduction of Fmax by soft tissue padding. Secondly, we decided that persons with osteoporosis have to wear hip protectors during the MA fall training. If participants wear hip protectors, the actual impact forces exerted on the femur will be substantially reduced. It has been shown that the best hip protectors reduce impact forces by between 65% and 85% [4,5]. According to the first safety criterion a fall was safe if the highest Fmax measured reduced by 10% for soft tissue padding and by 65% for the use of hip protectors, was lower than the threshold of 700 N (highest Fmax * 0.9 * 0.35 < 700 N).

For the second safety criterion we took into account that hip protectors are not always placed correctly with respect to the greater trochanter to optimally attenuate the impact forces [15,16]. In this second safety criterion we therefore left out the factor of force attenuation by hip protectors; the threshold was set at the average femoral fracture load for elderly minus one standard deviation (1900 N: 3100 N - 1200 N). Hence, the threshold was set at a value that should be safe for 84.1% of the elderly women if they did not wear hip protectors. According to the second safety criterion, a fall was safe if the highest Fmax measured, reduced by 10% for soft tissue padding, did not exceed the threshold of 1900 N (highest Fmax * 0.9 < 1900 N).

## Results

**Figure 2:**
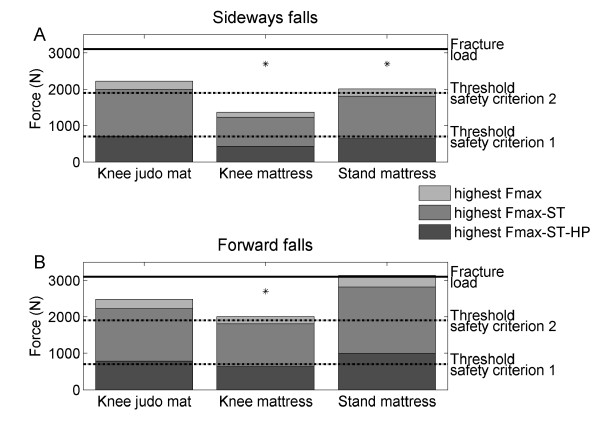
**Maximal hip impact forces for sideways and forward MA fall exercises**. The maximal hip impact force among all participants (highest Fmax) was corrected either for soft tissue padding (ST, 10% reduction) and wearing hip protectors (HP) that could attenuate the maximum hip impact force by at least 65% (safety criterion 1) or soft tissue padding only (10% reduction) (safety criterion 2). The corrected highest Fmax values were compared with thresholds of safety criterion 1 (700 N: average femoral fracture load for elderly minus two standard deviations) and safety criterion 2 (1900 N: average femoral fracture load for elderly minus one standard deviation), respectively. Asterisks (*) indicate fall conditions that met both safety criteria.

In general, the highest Fmax observed during the forward MA falls was higher than that found during the sideways falls under similar floor and fall height conditions (Table 1). Figure 2 shows the highest Fmax of all participants in the MA in sideways and forward falls from kneeling position on the judo mat and the thick mattress and from standing position on the thick mattress in relation to the thresholds of the two safety criteria.

**Table 1:** Descriptive statistics of the maximum hip impact force (Fmax) for the different fall conditions.

Fall exercise			Fmax (N)		
Fall direction	Fall height	Fall surface	Median	IQR	Max
Sideways	Knee	Judo mat	1769	407	2219
	Knee	Mattress	1330	131	1426
	Stand	Mattress	1551	439	2012
					
Forward	Knee	Judo mat	1745	624	2474
	Knee	Mattress	1453	669	2006
	Stand	Mattress	1833	419	3132

IQR = interquartile range, Max = highest Fmax.

For the sideways falls, the Fmax corrected for both soft tissue padding and hip protectors was lower than 700 N in all fall conditions. Hence, all the sideways falls met the first safety criterion (corrected Fmax was lower than 700 N). The Fmax if only corrected for soft tissue padding exceeded 1900 N in some of the participants during falls from kneeling position on the judo mat. Hence, the sideways falls from kneeling position on the judo mat did not meet the second safety criterion (range Fmax 1260-2219 N). If performed on the mattress, however, the sideways falls from kneeling position as well as from standing position met the second safety criterion (range Fmax 878-1426 N and 1216-2012 N, respectively)(Table 1, Figure 2).

For the forward falls, the falls from kneeling position did neither meet the first nor the second safety criterion if performed on the judo mat (range Fmax 1173-2474 N). However, if forward falls from kneeling position were performed on the mattress, Fmax met both safety criteria (range Fmax 1028-2006 N). Forward falls from standing position even when performed on the mattress did neither meet the first nor the second safety criterion (range Fmax 1389-3132 N)(Table 1, Figure 2).

## Discussion

This study determined whether MA fall training could be considered be safe for persons with osteoporosis as extrapolated from the data of young adults and using stringent safety criteria. The results showed that sideways falls from kneeling and standing position met the safety criteria if performed on a thick mattress. Forward falls only met the safety criteria if performed from kneeling position on the thick mattress. Hence, in order for the MA fall training to be safe for persons with osteoporosis, the fall training should be performed on a thick mattress and forward falls from a standing position should be excluded. In addition, participants should wear hip protectors that attenuate the maximum hip impact force by at least 65%. Specific data on the femoral fracture load of osteoporotic women have not been reported in the literature. Therefore, we based the safety criteria on the mean proximal femoral fracture load of elderly women (n = 28) with a mean age of 71 years (3100 SD 1200 N) [12]. It is very likely that at least some of these women had osteoporosis. Because of the strong correlation between fracture load and femoral neck BMD [12,17], the osteoporotic women were probably those with the lowest fracture loads. Fracture load is hard to estimate since it also depends on the loading rate [18] and direction of impact [19]. Therefore, we proposed a conservative first safety threshold of two standard deviations below the mean fracture load of elderly women, 700 N, which was lower than any individual femoral fracture load found in cadaveric studies [12,17-20]. For extra safety, we set a rather low threshold in the second safety criterion. In addition, the decision to include fall exercises was based on the highest Fmax observed among all participants. Hence, we think that our safety criteria are stringent enough to guarantee the safety of the included fall exercises for persons with mild to moderate osteoporosis.

In the present study, the safety of the MA fall training was determined only with respect to the risk for hip fractures. Falls may also result in other injuries, such as bruises, or head, arm and wrist injuries. The most important characteristics of the MA fall techniques are the rolling movement and head protection. To change the fall into a rolling movement, one should curve the trunk and neck. The trunk and neck flexion also prevent the head from impacting the ground. The risk of head impact is further reduced by slapping the arm to stop the rolling movement, which is another characteristic of the MA fall techniques. This arm slap is not believed to be harmful because the impact is distributed over a larger contact area due to the simultaneous impact of hand and forearm [8,11]. In previous studies with older healthy individuals, it was indeed not reported to be uncomfortable [10,11].

The safety of such MA fall training for persons with osteoporosis was recently confirmed by Smulders and coworkers [21]. Based on the results of the present study, the MA fall training of the original Nijmegen Falls Prevention Program [10] was modified. Thus far, 31 persons with osteoporosis (lowest T-score for proximal femur and lumbar vertebrae was between -4 and -2.5) participated and no injuries or adverse physical effects were reported during or after the training [21].

In experimental studies, MA fall techniques have been demonstrated to effectively reduce hip impact forces and, therefore, have the potential to reduce the hip fracture risk. MA fall techniques reduced the hip impact forces during a volitional fall by 12-27% when performed by experienced martial artists [7,8] and by 17% in young adults without previous experience in MA fall techniques after a 30-minute training session [9]. In addition, it was demonstrated that MA fall techniques were trainable in older individuals. After a five-session MA fall training, the fall performance improved and the hip impact force during a volitional fall was reduced by 8%. It was suggested that the MA fall training may have similar effects for hip fracture prevention as the prescription of bisphosphonates [11]. The effectiveness of MA fall techniques in reducing the hip impact forces are in line with the results of the biomechanical modeling study of Lo and coworkers [22]. That study revealed that a combination of knee flexion, waist flexion and trunk rotation is the most effective movement strategy to reduce the impact forces during a sideways fall (reduction of 56% compared to a 'broomstick' strategy). In addition, they found that this movement strategy was effective in reducing impact forces below the fracture load even when the effect of aging on muscle forces (reduction of 30% in muscle force) was simulated [22]. Since the combination of knee flexion, waist flexion and trunk rotation is characteristic of MA fall techniques to enable rolling after impact, the study of Lo and coworkers [22] confirms the potential beneficial effects of MA fall techniques for hip fracture prevention.

The effects of MA fall techniques on hip fracture risk in daily life, however, should be further investigated. A prerequisite for MA fall techniques to potentially contribute to hip fracture prevention in daily life, is the trainability of these techniques in the persons with osteoporosis. The results of a previous study on MA fall training in healthy elderly persons showed that they were indeed able to learn and apply these MA techniques during a volitional sideways fall from kneeling height. In addition, 15 of the 25 participants reported that they were also confident of being able to apply the MA fall techniques during an unexpected fall in daily life [11].

There is no conclusive evidence, however, for the applicability of the fall techniques in daily life, yet some indirect evidence is available. Although it is often suggested that a fall may happen too quickly to be able to select and execute a learned fall technique, the duration of a real-life fall from standing height has been reported to be 715 (SD 160) ms [23]. Given a voluntary reaction time of 180 ms for initiation a fall technique [24], there is some time to subsequently execute the fall technique before impact. The minimum movement time to execute the MA fall technique adequately was only 145-155 ms in young adults [24]. Although previous studies reported increased reaction times of 31-80 ms [23,25,26] and increased movement times for voluntary movements in the elderly [23], this probably still leaves sufficient time to select and execute a fall technique.

A limitation of the present study was that young adults participated instead of older persons to determine whether MA fall exercises could be safe for persons with osteoporosis. In general, the performance of fall exercises by older adults is expected to be less fluent than the performance by younger adults caused by a slower reaction time and poorer ability to coordinate muscle actions. This may result in higher hip impact forces. On the other hand, older adults are expected to have more fear of falling and are more cautious in their performance of the fall exercises, which presumably results in lower impact velocities and, consequently, lower hip impact forces.

Another limitation was the small sample size in the present study. It may not represent the normal variability in the normal population. Because it is likely that heavier and/or taller persons experience higher hip impact forces during a fall, it could affect the decision whether the fall exercises of the MA fall training is safe or not in the present study. On the other hand, older age and a low body mass (rather than a high body mass) are the most important risk and screening factors for osteoporosis [27,28] and are used to predict bone mineral density (T-score) [29]. It indicates that heavier persons have stronger bones. In addition, it is expected that heavier persons have a thicker soft tissue layer overlying the greater trochanter of the femur that can absorb energy during hip impact. Increased soft-tissue thickness is strongly correlated with decreased peak femoral impact force [14]. We, therefore, believe that the fall exercises that we identified as safe in the present study are also safe for heavier persons who may experience higher hip impact forces, but also have more soft tissue padding and stronger bones than the bone strength as used in the safety criteria.

## Conclusions

Based on the data of young adults and stringent safety criteria, the MA fall training was expected to be safe for persons with osteoporosis if they wear hip protectors that could attenuate the maximum hip impact force by at least 65% during the training, perform fall exercises on a thick mattress, and avoid forward fall exercises from a standing position. Since MA techniques reduce hip impact forces and can be learned by older persons, MA fall training may prevent hip fractures among persons with osteoporosis.

## Competing interests

The authors declare that they have no competing interests.

## Authors' contributions

BG, VW and WL conceived of and designed the study. BG and VW recruited the participants and performed the measurements. BG performed the analysis and made the first draft of the manuscript. ES, VW and JD contributed to the analysis and interpretation of the data. ES, JD, WL and VW revised the manuscript critically, and all authors read and approved the final manuscript.
